# Structural adaptations of aboveground vegetative organs to cangas: A case study with the Amazonian species *Carajasia cangae* R.M.Salas, E.L.Cabral & Dessein (Rubiaceae), which grows on ferruginous outcrops

**DOI:** 10.1371/journal.pone.0354749

**Published:** 2026-08-21

**Authors:** Kleber Resende Silva, Daniela Boanares, Mônica Lanzoni Rossi, Joana Patrícia Pantoja Serrão Filgueira, Adriana Pinheiro Martinelli, Carolina Carvalho, Cecílio Frois Caldeira, Mauricio Takashi Coutinho Watanabe

**Affiliations:** 1 Vale Institute of Technology, Belém, Brazil; 2 Center of Nuclear Energy in Agriculture, University of Sao Paulo, Piracicaba, São Paulo, Brazil; Universidade Federal de Alfenas, BRAZIL

## Abstract

The Brazilian ironstone outcrops (cangas) impose challenging conditions for establishing species. Adapted species should exhibit different responses to xeric conditions of cangas. To understand these adaptations, our plant model was *Carajasia cangae* R.M.Salas, E.L.Cabral & Dessein, an endemic and threatened species restricted to the cangas of the Amazon Forest. Herein, we investigate structural adaptations that are important for its conservation, including anatomical characteristics of stems and leaves. To this goal, we evaluated the plant anatomy, including developmental aspects, between plants from the natural environment (*in situ*) and cultivated specimens (*ex situ*), providing information on phenotypic plasticity. Developmental aspects and the effects of seasonality on leaf anatomy and ultrastructure were also analyzed. Under natural conditions, the plants exhibit rigid stems with secondary growth and leaves with a thicker blade and mesophyll. In these plants, mucilaginous cells are associated with the vascular bundles. Such characteristics, however, do not occur in the individuals under cultivation, which are fragile and etiolated. The leaf structure, in particular, is well adapted to the high radiation of the cangas. This condition is related to the rapid development of epidermal and mesophyll characteristics during regrowth in the rainy season. Seasonality modulates the arrangement of the mesophyll and the synthesis of phenolic compounds. In the dry season, the fibrous vascular bundles are more lignified, contributing to the support of the leaves, including senescent ones. The ultrastructure reveals that subcellular dissolution of the mesophyll occurs during leaf death. The results showed that *C. cangae* presents little adaptability to the cultivation conditions tested and that its life cycle is mediated by seasonality. Therefore, the integrity of the canga areas where the species occurs must be maintained for its conservation, or its relocation must be made to areas with similar soil and climate conditions.

## Introduction

Lineages that occur naturally in limiting environments are adapted to severe conditions, such as high temperature and radiation, periods of drought, and shallow soils and/or with few nutrients available. Plants growing under these xeric conditions exhibit different anatomical responses, such as stomatal closure that reduces transpiration or differentiation of covering tissues and cuticle that reduces aboveground organ permeability, and accumulation of solutes with antioxidant activity within idioblasts [1–3]. The Brazilian flora comprises many drought-tolerant species that can serve as models, exhibiting diverse adaptive strategies. Some extreme responses, such as desiccation tolerance (e.g., resurrection plants) to prolonged drought, rely on morphoanatomical and ultrastructural mechanisms that minimize mechanical stress during dehydration and allow rapid recovery upon rehydration (e.g., [4–6]). Nevertheless, because this strategy entails high morphophysiological and metabolic costs [5], many seasonally adapted plants instead invest in drought-avoidance strategies, such as regrowth and/or seed germination during the rainy season (e.g., [7,8]). Eventually, these plants must develop structural adjustments associated with drought tolerance, especially in xeric environments, such as the rocky outcrops of rupestrian grasslands (e.g., campos rupestres, rupestrian or rocky fields, rupestrian savannas, and cangas), which are ancient ecosystems with a high richness of herbaceous and subshrub endemic species [9–11].

The cangas, the Brazilian iron outcrops, are banded iron formations that mainly cover the Iron Quadrangle, in the state of Minas Gerais, and the Serra dos Carajás, in the eastern portion of the Amazon Rainforest, in the state of Pará [9,12–15]. The ferruginous outcrops of the cangas are related to high biodiversity [13,15], with different plant lineages tolerant to heavy metals (i.e., edaphic endemism; 11) and capable of surviving seasonal drought periods [12,13]. In a comparative context with other rupestrian grasslands, little is known about the plant communities associated with iron-rich outcrops [12] and adaptive plant mechanisms, including anatomical responses.

Considering structural responses, few studies have evaluated the adaptive aspects of plants in the Amazonian cangas. Recently, Silva et al. [16] showed that two species of *Ipomoea* L. (Convolvulaceae) growing on canga exhibit leaf plasticity when compared to natural conditions (*in situ*; xeric conditions) and cultivation (*ex situ*; mesic conditions). Under natural conditions, the leaves have smaller and numerous stomata, thick cuticles, mesophyll with developed palisade parenchyma, and cells storing phenolic compounds and mucilage [16]. These characteristics were discussed as responses to high light and low water supply. On the other hand, under *ex situ* conditions, the leaves were covered by a thin cuticle, and stomata, trichomes, and idioblasts containing calcium oxalate crystals were less frequent [16]; these adjustments were favorable for the growth of specimens in conditions different from those imposed by the natural environment. In this context, studies comparing different growth conditions can demonstrate the phenotypic plasticity of the species, providing adaptive evidence [16–18]. Indicating whether a species exhibits phenotypic plasticity may be important for mediating management actions for vulnerable taxa, evaluating the possibility of reintroduction into new areas (see 16).

*Carajasia cangae* R.M.Salas, E.L.Cabral & Dessein (Rubiaceae), our study plant model, is a monotypic species, endemic and restricted to some ferruginous rocky outcrops of the National Forest of Carajás (FLONA de Carajás) [19]. Its conservation is considered a priority due to its small population size [20] and restricted occurrence in a region with mining activities [21]. Small (young recruits) and large individuals (plants at least biennial) co-occur during the rainy season, producing flowers, fruits, and seeds [8]. While small individuals establish from seeds and complete a short life cycle during the rainy season [8], some larger ones, with more branches, can persist across seasons. It suggests that larger individuals have developed strategies to tolerate seasonal drought. In this context, the anatomy of the species and its adaptive traits remain unexplored, despite their importance for understanding its biology. Moreover, seasonally modulated structural responses have not yet been investigated in Amazonian canga plants. In light of climate change, studies assessing the strategies of vegetative organs under water stress are particularly encouraged.

The main objective of this study is to elucidate structural responses related to the aboveground organs of *C. cangae* specimens in the canga environment. We carried out comparative analyses between plants under natural and cultivated conditions; we believe that in cultivated conditions, the vegetative anatomy will be quite distinct, allowing the identification of structural characteristics related to the xeric conditions of the cangas. Considering the regrowth capacity of the species, basal portions of the stem and nodes (where the axillary buds occur) were analyzed; this provides insights into organ development during the rainy season. In relation to seasonality and its impacts on leaf structure, tissue, and ultrastructural adjustments, understanding leaf dynamics during dry and rainy periods is crucial. These results are significant for mediating species conservation actions.

## Materials and methods

### Study area and botanical material

Field observations and sample collection were conducted over the period from 2022 to 2023 in canga areas in Serra Sul (canga plateau S11; −50.424934, −6.341843; *Viana et al. 5263* deposited in BHCB) of the FLONA de Carajás; the collection permit for aboveground vegetative organs was promoted by Sistema de Autorização e Informação em Biodiversidade (SISBIO, number 76784–1). The climate in the region is classified as humid tropical – Aw according to the Köppen system – with a dry season from June to September (average precipitation of 32 mm) and a rainy season from November to April (average precipitation of 248 mm), and average monthly temperatures vary between 19–31°C [22].

**Fig 1:**
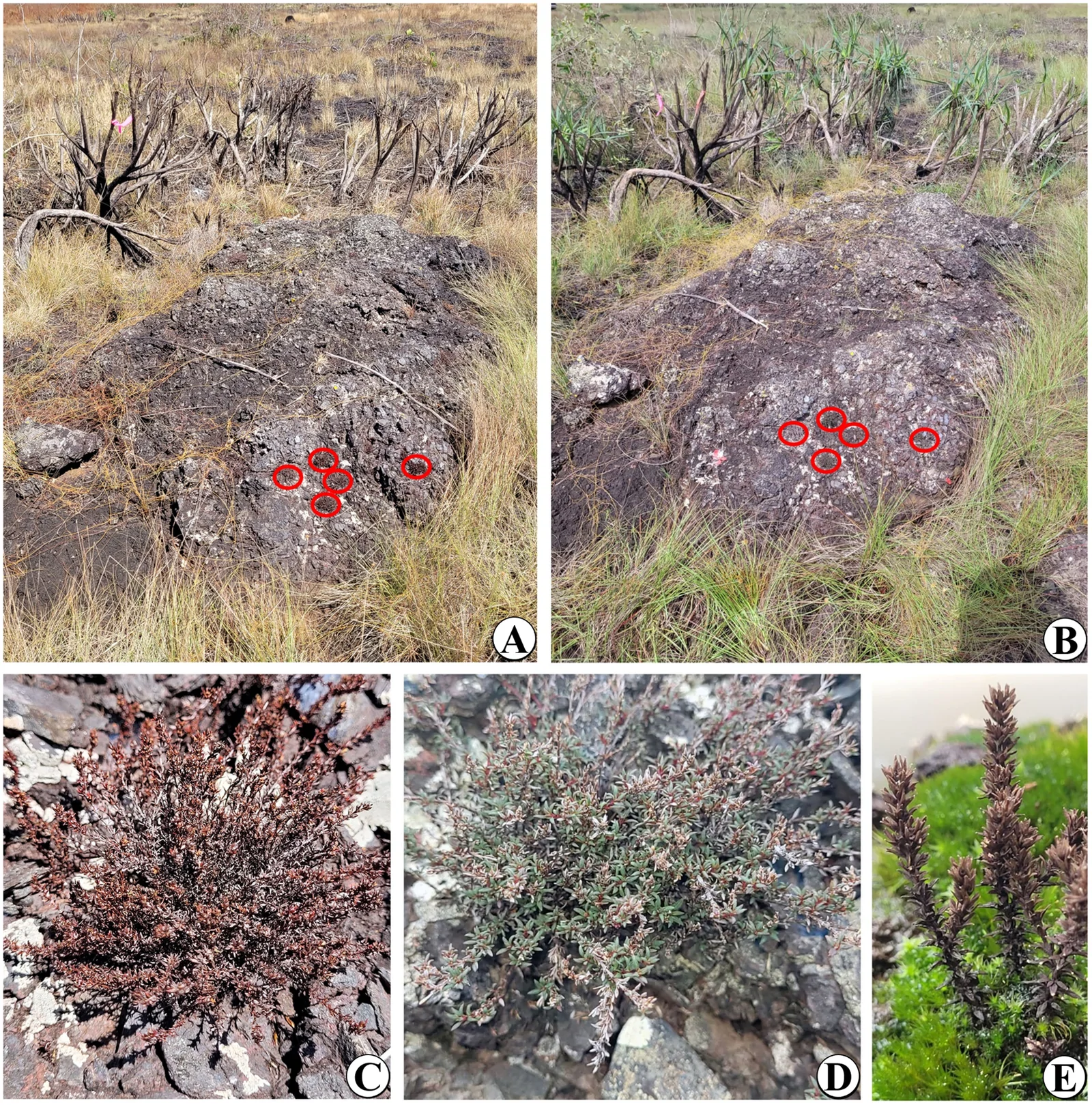
Habit and morphology of *Carajasia cangae* on the canga outcrops. A-B. Individuals (red circles) on rocky outcrops, in the dry and rainy seasons, respectively. C-D. Individuals in the dry and rainy seasons, respectively. E. Dead individual after the dry season, with leaves attached to the stem axes.

*Carajasia cangae* was first described nearly a decade ago by Salas et al. [19] as a small (2–10 cm tall), herbaceous lithophyte (Fig 1A–D). The species has an erect habit with tetragonal, tiny (1.8–2.1 × 0.5–0.7 mm), and vinaceous branches; glabrous, pseudoverticillate leaves with a conspicuous midrib. Larger individuals of *C. cangae* are drought-resistant (Fig 1A, C) and regrow during the rainy season (Fig 1B, D). However, some individuals die but retain their leaves attached to the stem (Fig 1E).

### Experimental design: natural environment and cultivation

For data collection under natural conditions (radiation >1000 mmol m^-2^ s^-1^), larger individuals (ca. 10 cm tall; n = 3) were selected in the non-reproductive stage. These specimens were collected during the rainy season for comparison with specimens under cultivation in the laboratory without water restriction. Later in the dry season, leaf samples from the same individuals in the natural environment were also collected to compare the effects of seasonality.

To standardize and compare the morphoanatomy of plants from the natural environment with those grown under cultivation conditions, we also selected cultivated specimens (n = 3) that reached approximately 10 cm in length (maximum size obtained in the laboratory). To this purpose, we harvested seeds from their natural habitat (canga plateau S11). The seeds exhibit orthodox behavior, being released during the dry season, with moderate viability (approximately six months) [8]. In the present study, we used seeds (n = 20) that underwent three months of field storage during the dry season, as this was the most effective condition tested for alleviating seed dormancy, resulting in higher germination rates and speed (see 8). Germination was carried out on germitest paper within Petri dishes and conducted within a plant growth chamber set to a day: night temperature regime of 28:22°C, relative air humidity maintained at 65%, and a photoperiod of 12:12h with photosynthetic active radiation of 100 µmol m^-2^ s^-1^. Following germination, seedlings (n = 10) were transplanted into canga topsoil collected from occurrence areas of *C. cangae*, where the seeds were initially harvested; each seedling was kept individually in 0.27 dm^3^ pots. These seedlings were cultivated for 10 months under the same environmental conditions as during germination. Water loss through evapotranspiration was replaced daily by distilled water. It should be noted that few seedlings and young plants survived under laboratory culture conditions. Due to the difficulty in obtaining *C. cangae* plants (ex situ), only one branch (stem) and 1–2 leaves were removed from each individual to minimize the impacts on their survival. Likewise, we did not include root anatomy to minimize impacts on specimen survival.

Small stem fragments (nodes and internodes) were obtained from already differentiated regions, approximately 2 cm below the stem apex, where the leaves were already fully expanded. The fully expanded leaves were also collected. The nodes were analyzed to characterize vegetative buds and short lateral branches to provide data on the development of aerial parts. Additional analyses were also carried out at the base of the stem in the samples from natural environments. All samples were fixed in FAA 70 (37% formaldehyde, glacial acetic acid, 70% ethanol, 1:1:18 v/v; [23]) and stored in 70% ethanol for morphological and anatomical studies. Moreover, to investigate leaf ultrastructure, the samples collected from plants in the dry and rainy seasons were fixed in a modified Karnovsky solution (2.5% glutaraldehyde; 2% paraformaldehyde, 0.1 M phosphate buffer, pH 7.2; [24]) and maintained under refrigeration.

### Plant morphology

Fragments of branches with leaves already stored in 70% ethanol were previously observed under a stereomicroscope (Zeiss, Oberkochen, Germany, SteREO Discovery V12) with images obtained by an attached camera (Zeiss, AxioCam 712 colour) and ZEN 3.4 (blue edition) software. These results were obtained to characterize general aspects of plants in natural environments and under cultivation.

### Light microscopy (LM) and scanning electron microscopy (SEM) of plants under natural and cultivation conditions

Samples previously stored in 70% ethanol were used for LM and SEM studies. For LM, the samples were dehydrated in n-butyl alcohol series and embedded in 2-hydroxyethyl-methacrylate (Leica Historesin Embedding Kit) [25]. With a rotary microtome (Leica, RM 2255), cross and longitudinal sections (4–6 µm thick) of the stem (nodes and internodes) and the middle region of the blade were obtained and placed on slides. The sections were stained with periodic acid–Schiff’s reagent (PAS) and toluidine blue [26–28] and mounted with Entellan (Merck). The slides were examined using a light microscope (Zeiss, Axio Scope A1) with an attached camera (Zeiss, AxioCam ICc 5) and AxioVision (Version 4.8.3.0) software.

Histochemical tests were performed with ferric chloride to confirm the presence of phenolic compounds [23], sudan III for lipids (cuticle) [29], and hydrochloric acid and glacial acetic acid solutions for calcium oxalate detection [30]. Observation of starch grains was facilitated by PAS staining [28].

Since anatomical characterization revealed trichomes and papillae in aerial organs (see Results), SEM study was performed for micromorphological characterization. Thus, in order to illustrate these structures, leaves under natural conditions were dehydrated in an ethanol series (70–100%), critical point dried in CO_2_ (Balzers CPD 050), coated with gold (Bal-Tec SCD 050), and examined in a Zeiss LEO 435VP SEM, at 20 kV. Images were created using the SEM LEO Interface User digital imaging system.

### Light microscopy (LM) and transmission electron microscopy (TEM) of leaves in the dry and rainy seasons

Samples of leaves stored in the Karnovsky solution were rinsed in phosphate buffer (0.1 M) and post-fixed in osmium tetroxide (1% in 0.1 M phosphate buffer, pH 7.2) for 1 h at room temperature (20–25 °C). After that, the samples were dehydrated in a graded acetone series (30%–100%) and embedded in Spurr [31] low-viscosity resin (EMS, Electron Microscopy Sciences, Hatfield, PA) for 48 h at 70 ºC. For prior analyses, cross semi-thin sections (120–200 nm) were obtained at the middle third of the blade; these sections were stained with 2.5% toluidine blue in water. The ML analysis was conducted using a light microscope with a camera attached (with the same settings described previously).

Subsequently, TEM studies were carried out with ultra-thin sections (60–90 nm) obtained from the mesophyll at the adaxial surface (palisade chlorenchyma) using a diamond knife in an ultramicrotome (Porter Blum MT2, Dupont-Sorvall), collected on copper grids (300 mesh) and post-stained with 2.5% aqueous uranyl acetate, followed by 0.1% lead citrate [32]. The sections were examined at 80 kV under a transmission electron microscope (JEM1400 JEOL), obtaining electron micrographs.

### Quantitative analysis

From the anatomical sections obtained for each treatment (cultivation, natural environment-rainy season, and natural environment-dry season), the thickness (in μm) of the leaf blade, mesophyll, epidermis (adaxial and abaxial surfaces), and palisade and spongy parenchyma was measured in regions between the margin and the midrib. For each treatment, three leaves were analyzed, with ten measurements taken per leaf, resulting in 30 measurements per tissue or leaf region per treatment. Measurements were performed using ImageJ software (National Institutes of Health, Bethesda, MD, USA), calibrated with the scale bars of the micrographs. Means and standard deviations were calculated for all variables. All data analyses were conducted in the R environment [33]. Data normality was verified with the Shapiro–Wilk test (shapiro.test function), and mean comparisons were performed using one-way analysis of variance (ANOVA), followed by post hoc Tukey HSD test. A comparative Fig of the three treatments was generated using the ggplot2 package. In the Results section, cultivated leaves were first compared with those from the natural environment during the rainy season (see Growth conditions modify the anatomy of aboveground organs), followed by comparisons between seasons (see Seasonality modifies the anatomy and ultrastructure of leaves).

## Results

### Morphological aspects

**Fig 2:**
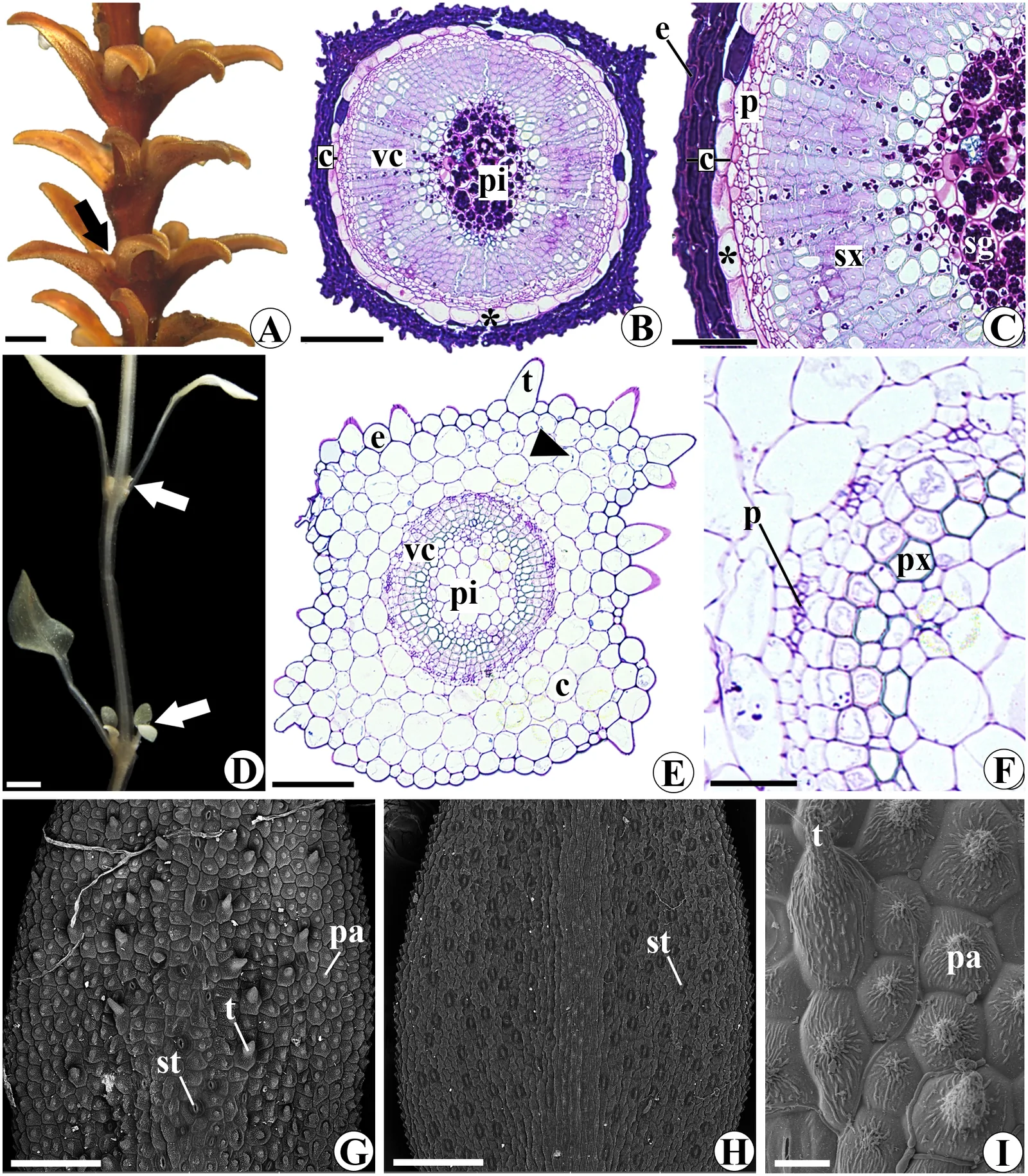
Stem structure and leaf morphology of *Carajasia cangae*, under natural (A-C, G-I) and cultivation conditions (D-F). A, D. Details of the branches showing the nodes, internodes, and short lateral branches (samples stored in 70% ethanol). B, E. General anatomical aspects of the stem, in cross-sections; note the secondary growth under natural conditions (B). C, F. Details of the stem anatomy, in cross-sections. G-H. General aspects of the adaxial and abaxial leaf surfaces, respectively, under scanning electron microscopy (SEM). I. Trichomes and epidermal papillae on the adaxial surface of the blade, under SEM. Arrows, short lateral branches; arrowhead, chloroplasts; c, cortex; e, epidermis; p, phloem; pa, papilla; pi, pith; px, primary xylem; sg, starch grains; sx, secondary xylem; t, trichome; vc, vascular cylinder; *, innermost layer of the periderm. Scale bars: A: 0.5 mm; B, E: 100 μm; C: 50 μm; D: 1 mm, F: 25 μm; G-H: 200 μm; I: 20 μm.

Plants growing in natural environments (i.e., canga outcrops) are quite different from those grown under laboratory conditions, despite *C. cangae* stands out for its small size, with leaves and branches also reduced, with one pair of leaves per node (Fig 2A, 2D). In the canga outcrops, these plants exhibit more robust and rigid stems with reduced internodes (Fig 2A). Axillary buds at each stem node form new, very reduced lateral branches (Fig 2A – arrow). On the other hand, plants under cultivation exhibit delicate and etiolated stems and leaves; note the translucent stems with elongated internodes, as well as the leaves, which also have a wider blade (Fig 2D). It is worth noting that the plants in the laboratory grew much faster, possibly associated with light stress and etiolation. Axillary buds developing into new reduced lateral branches are also observed under cultivation conditions (Fig 2D – arrows). Trichomes (longer) and epidermal papillae (shorter) occur on aerial organs (Fig 2E, 2G, 2I) in both growth conditions. However, they are less noticeable on the stem of specimens growing under natural conditions since the epidermis becomes collapsed (see anatomical description). When comparing the leaf surfaces, the trichomes and epidermal papillae are abundant on the adaxial side (Fig 2G-2H). Both trichomes and papillae are covered by striated cuticle (Fig 2I).

### Growth conditions modify the anatomy of aboveground organs

Plants of *C. cangae* grown under natural conditions (rainy season) have stems and leaves with distinct anatomical structures from those grown under cultivated conditions. The stem, in cross-section, exhibits a tetragonal outline but with convex surfaces (Fig 2B) due to secondary growth; note that secondary xylem formation occurs (Fig 2B-2C). As a consequence of the secondary growth, the epidermis and cortex are collapsed, with periclinally elongated cells storing phenolic compounds (Fig 2B-2C); only the innermost layer of the cortex has larger cells (Fig 2B-2C – asterisks). The secondary xylem is differentiated into vascular elements, fibers, and parenchymatous rays (Fig 2C). Phloem tissues are less developed than xylem (Fig 2B-2C), even though they show more conductive cells in canga plants (Fig 2C) than in cultivated plants (Fig 2F). Vascular cambium is not evident, indicating a complete differentiation of secondary tissues. A parenchymatous pith stands out with rounded and large cells storing many starch grains (purple grains by PAS staining) (Fig 2B-2C). Under cultivation, the etiolated stem, in cross-section, exhibits a distinct tetragonal outline with a primary structure or with a secondary xylem poorly developed (Fig 2E-2F). The epidermis is single-layered, with cells of different sizes, including papillose cells and trichomes (Fig 2E). The cortex is composed of three to four layers of rounded cells, with those close to the epidermis having chloroplasts (Fig 2E – arrowhead). The starch grains were not observed in cultivated plants (Fig 2E).

**Fig 3:**
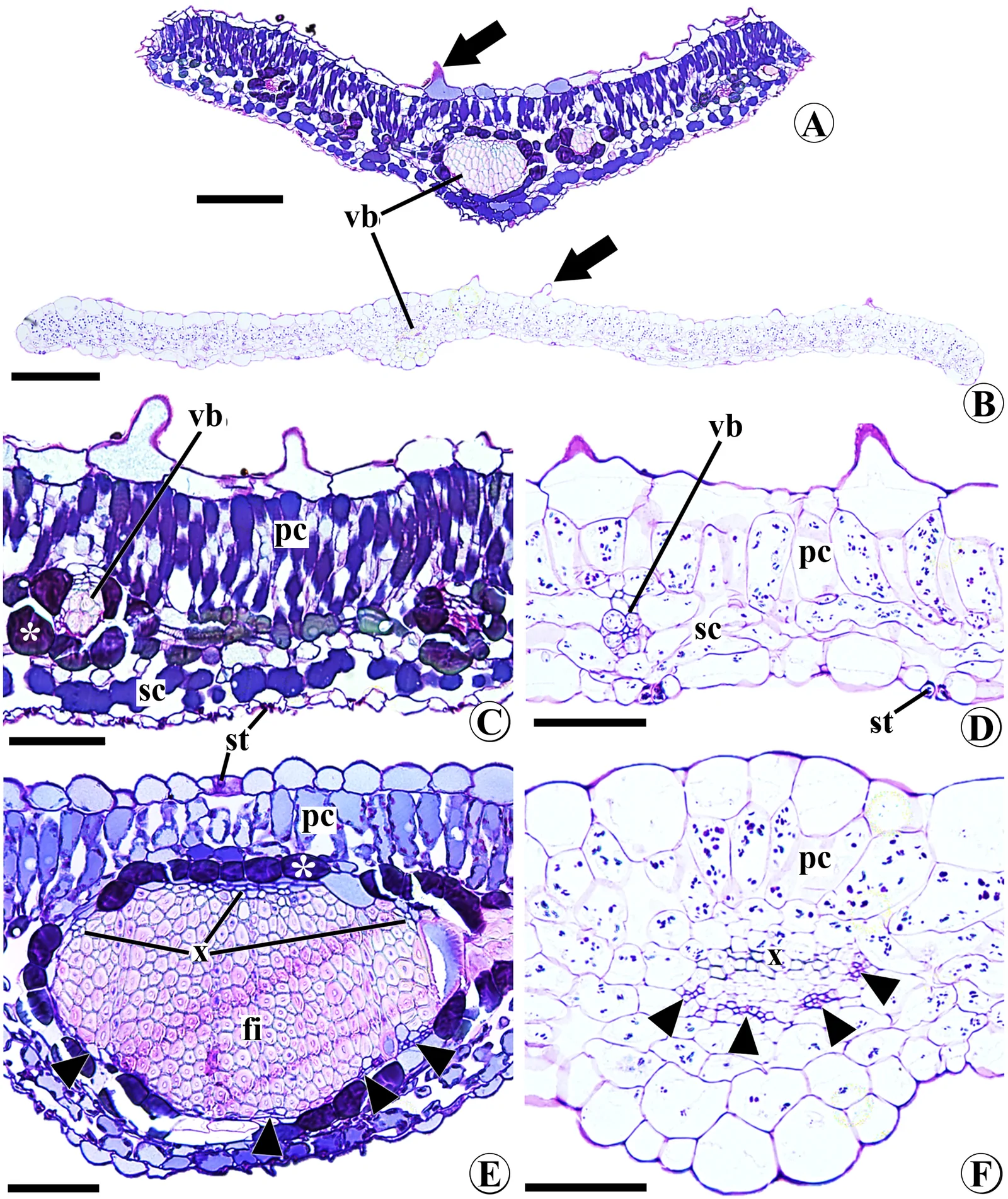
Leaf anatomy of *Carajasia cangae*, in cross-sections, under natural (A, C, E) and cultivation conditions (B, D, F). A-B. General aspects of the blades. C-D. Details showing epidermis, mesophyll, and small vascular bundles. E-F. Details of the midribs. Arrows, trichomes; arrowheads, phloem; fi, fibers; pc, palisade chlorenchyma; sc, spongy chlorenchyma; st, stomata; vb, vascular bundles; x, xylem; *, mucilaginous cells. Scale bars: A: 100 μm; B: 200 μm; C-F: 50 μm.

**Fig 4:**
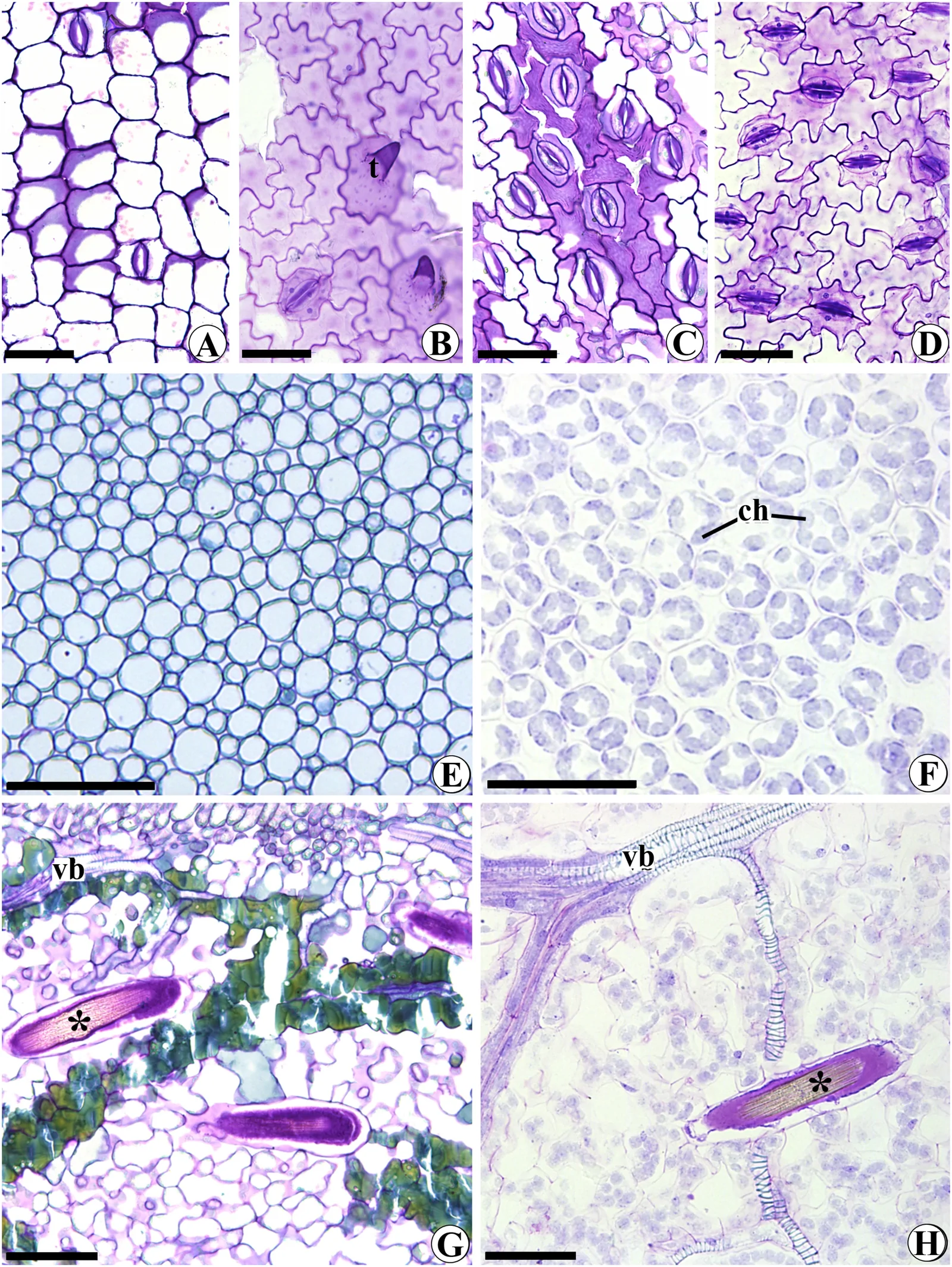
Leaf anatomical details of *Carajasia cangae*, in frontal view (A-D) and longitudinal sections (E-H), under natural (A, C, E, G) and cultivation conditions (B, D, F, H). A-B. Epidermis on the adaxial surface. C-D. Epidermis on the abaxial surface. E-F. Palisade chlorenchyma with more cells in canga leaves (E), but with larger chloroplasts in cultivated plants (F). G-H. Spongy chlorenchyma; note that cells storing phenolic compounds only occur under natural conditions (G). ch, chloroplasts, t, trichome; vb, vascular bundles; *, raphide-containing idioblasts. Scale bars: A-H: 50 μm.

**Fig 5:**
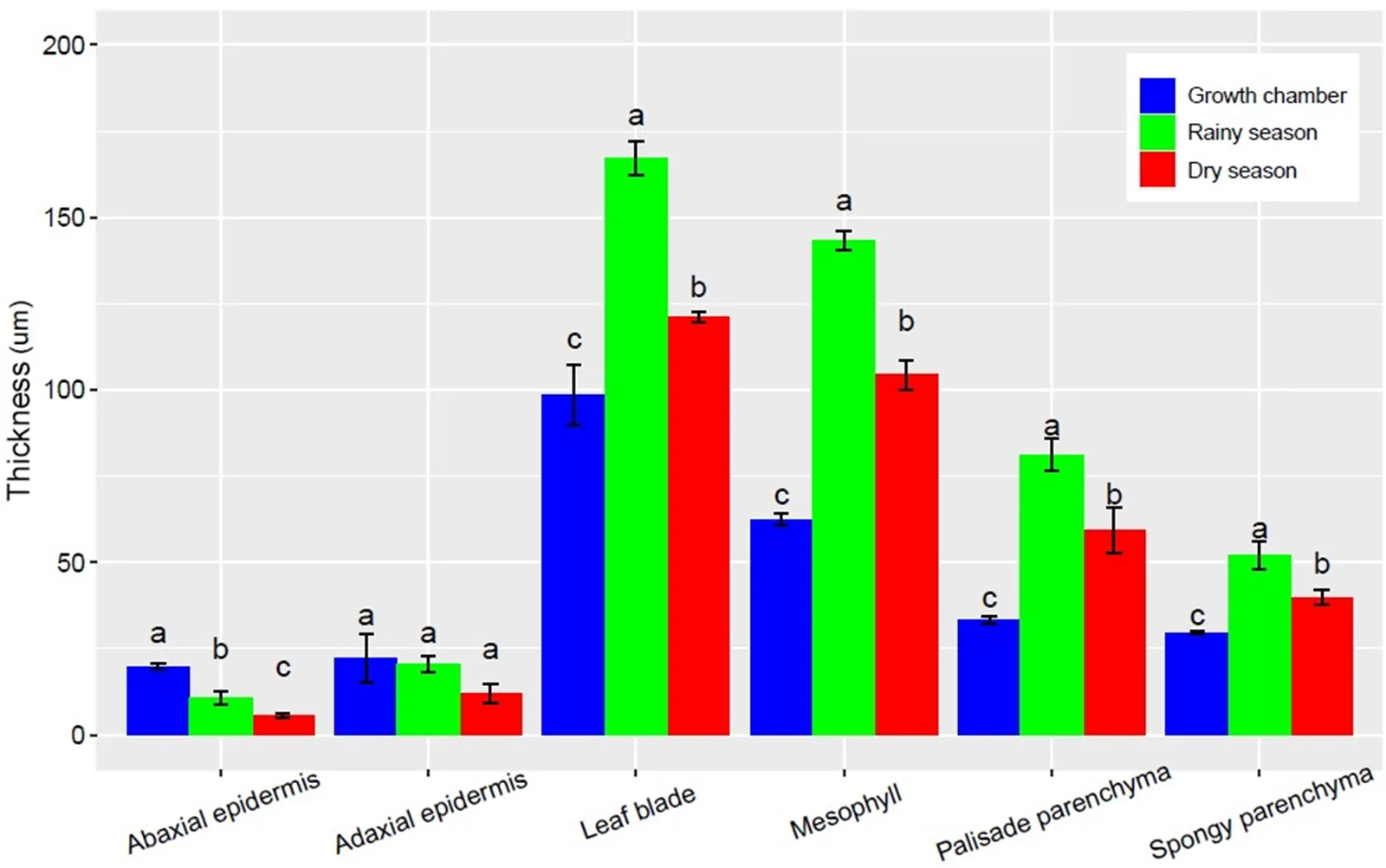
Leaf anatomical traits of *Carajasia cangae* plants grown under cultivation and in the natural habitat during the rainy and dry seasons. For each trait, different letters indicate statistically significant differences between means according to Tukey’s test (P < 0.05). Bars represent standard deviations (n = 30 per treatment).

In cross-sections, leaves of *C. cangae* in both treatments exhibit a single-layered epidermis, dorsiventral mesophyll, and collateral vascular bundles (Fig 3A-3F). In the epidermis, trichomes and papillae predominate on the adaxial surfaces (Fig 3A-3D). Stomata are located at the same level as the other epidermal cells (Fig 3C-3D), are of the paracytic type, and predominate on the abaxial surfaces (Figs 2H and 4C-4D). On the adaxial surfaces, stomata are sparse (Figs 2G, 3E, and 4A-4B). Despite these similarities, leaves expanding under natural conditions are visually smaller (Fig 2A) with thicker blades (Figs 3A and Fig 5 – leaf blade).

In frontal view, the leaf epidermis on the adaxial surface of individuals from the natural environment has quadrangular to rectangular cells with straight anticlinal walls (Figs 2I and 4A). In contrast, in cultivated plants, the epidermal cells have sinuous walls (Fig 4B). On the abaxial surface, the sinuous contour of the cells is maintained in both treatments (Fig 4C-4D). In plants from the natural environment, epidermal cells appear larger on the adaxial surface than on the abaxial surface (Fig 3A, 3C, 3E). In contrast, cultivated leaves exhibit cells that are visually similar in size on both surfaces, or they are slightly smaller on the abaxial surface (Fig 3B, 3D, 3F). When treatments are compared, the abaxial epidermis of cultivated leaves is the thickest (Fig 5 – abaxial epidermis), with noticeably larger cells; the adaxial surface does not statistically differ between treatments (Fig 5 – adaxial epidermis).

The mesophyll of leaves in the natural environment is thicker (Fig 5 – mesophyll) due to more elongated palisade cells (Fig 5 – palisade parenchyma) and a thicker spongy chlorenchyma (Fig 5 – spongy parenchyma). In cultivated plants, although the palisade parenchyma is less thick (Fig 5 – palisade parenchyma), its cells are voluminous (Fig 3D). Visually, the mesophyll also differentiates into more numerous and smaller cells under natural conditions (Figs 3C and 4E) than those under cultivation conditions (Figs 3D and 4F); in the latter, the chloroplasts are conspicuous (Fig 4F). The vascular bundle of the midrib under natural conditions is conspicuous and fibrous (Fig 3A, 3E) than in cultivated leaves (Fig 3B, 3F).

The synthesis of phenolic compounds in the leaves occurs only under natural conditions; these compounds are stored in epidermal cells, mesophyll, and in the vascular sheath (blue-green colored cells; Figs 3A, 3C, 3E, and 4G). The cells of the vascular sheath, in addition to phenolic compounds, also store polysaccharides (mucilaginous content), which react to PAS, giving a pronounced coloration (Fig 3C, 3E – asterisks). Under both growth conditions, idioblasts containing raphides (calcium oxalate crystals) and mucilage are observed in the leaves (Fig 4G-4H).

### Aspects of shoot development and regrowth

**Fig 6:**
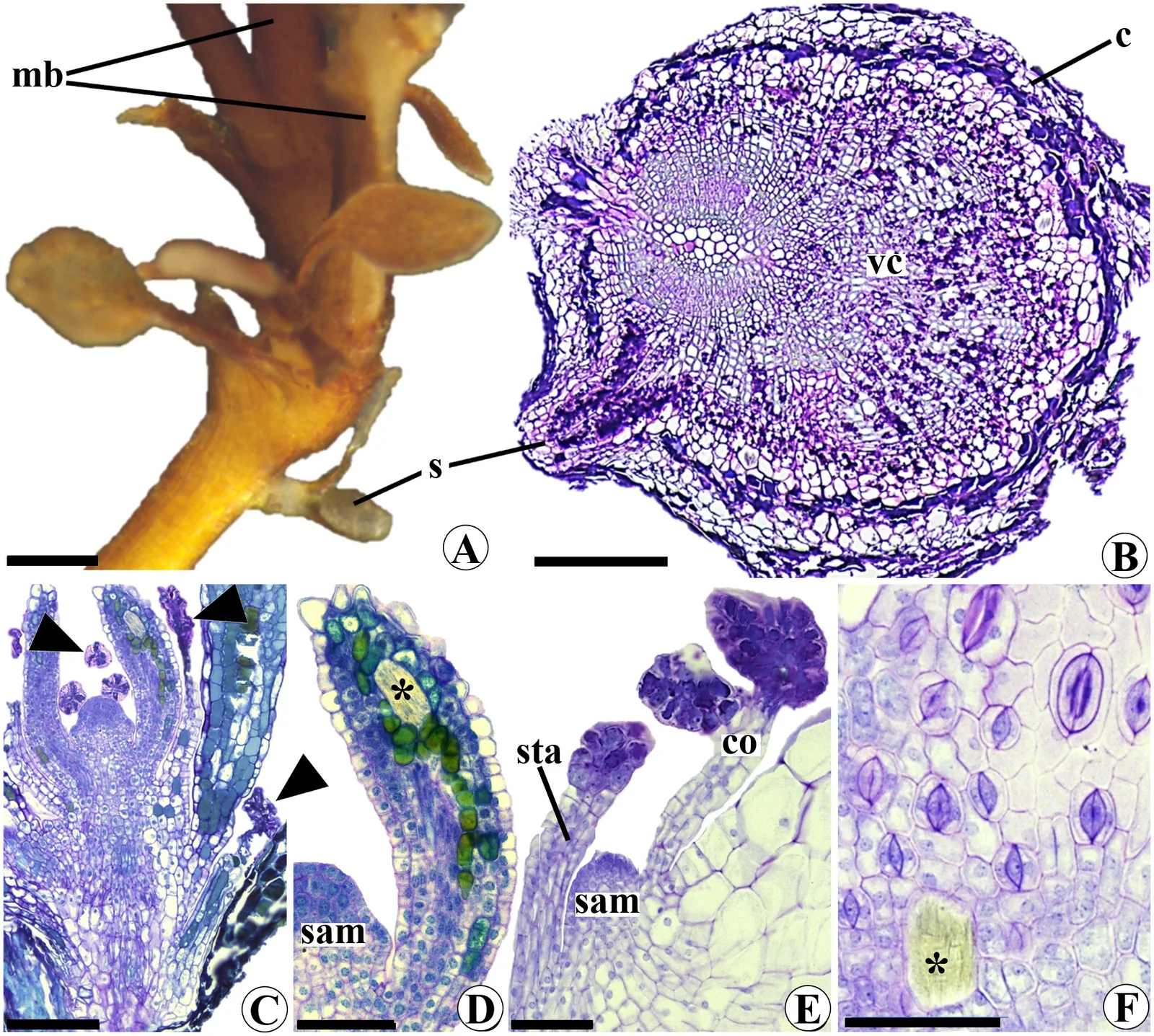
Structure of the stem base and reduced lateral branches of *Carajasia cangae*, under natural (A-D) and cultivation conditions (E-F). A-B. Morphology and anatomy, in cross-section, of the stem base, respectively. C. General aspect of the short lateral branch, in longitudinal section. D-E. Details of the apex of the short lateral branches, in longitudinal sections. F. Stomata development and part of the mesophyll on the abaxial surface, in longitudinal section. Arrowheads and co, colleters; c, cortex; mb, main branches; s, sprout; sam, shoot apical meristem; sta, stalk; vc, vascular cylinder; *, raphide-containing idioblasts. Scale bars: A: 0,5 mm; B: 200 μm; C: 100 μm; D-E: 50 μm; F: 25 μm.

Material collected in the rainy season showed signs of regrowth. Stem sprouts and the main branches are inserted at the base (Fig 6A-6B), as well as adventitious roots; this region is partially covered by soil, which may cover the vegetative buds (i.e., bud burial) and small sprouts. Anatomically, this region is wide, with a well-developed secondary structure (Fig 6B), mainly the xylem; starch grains were observed in the parenchymatous cells of the vascular tissues.

Regrowth also occurs on the reduced lateral branches, with new leaves developing (Fig 6C-6D). We compared these results with those from cultivated plants (Fig 6E-6F). Colleters occur in the leaf axils and close to the shoot apical meristem (SAM) (Fig 6C, 6E). The Colleters are simple or branched, non-vascularized, with the glandular portion composed of cells with a dense cytoplasm (Fig 6E). In older colleters, the glandular cells may be collapsed, while the parenchymatous cells of the stalk store phenolic compounds. In these reduced branches, we observed some leaf differentiation aspects. In these expanding leaves, both in plants from natural environments and in cultivated plants, differentiation of epidermal papillae (Fig 6D) and trichomes, stomata (Fig 6F), and calcium oxalate idioblasts (Fig 6D, 6F – asterisks) occurs. In frontal view, the abaxial surface of the primordia and young leaves, the epidermis still presents cells with straight or slightly sinuous walls (Fig 6F). In samples under natural conditions, the differentiation of leaf cells containing phenolic compounds is still observed (Fig 6C-6D).

### Seasonality modifies the anatomy and ultrastructure of leaves

**Fig 7:**
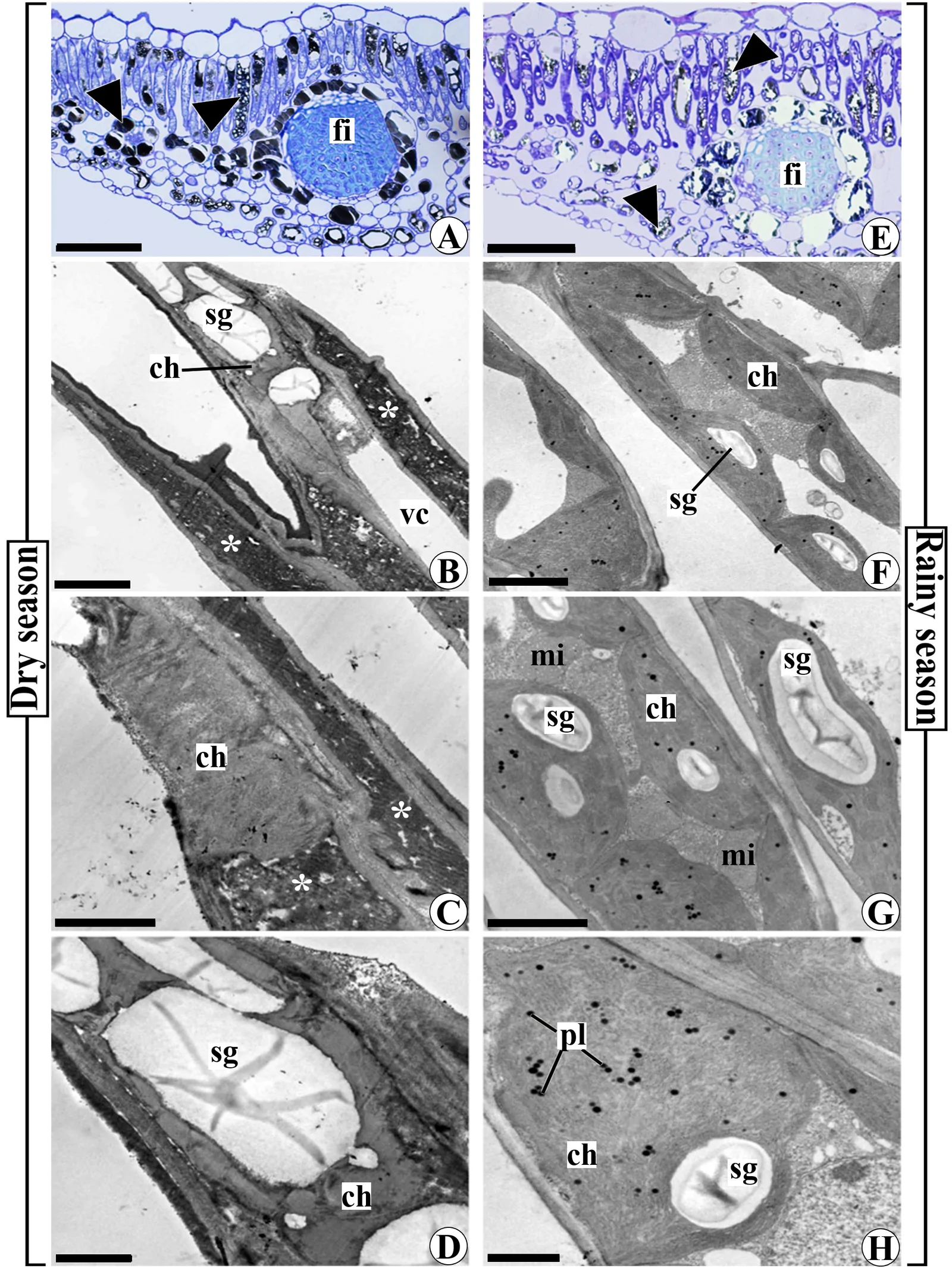
Leaf anatomy and ultrastructure of *Carajasia cangae* in the dry (A-D) and rainy (E-H) seasons, in cross-sections (A, E) and under transmission electron microscopy (TEM) (B-D, F-H). A, E. Details showing epidermis, mesophyll, and midrib. B-D. Subcellular dissolution, including degradation of chloroplasts. F-H. Functional cells with intact membranes and organelles; note the elongated chloroplasts (F-G). Arrowheads, cells storing phenolic compounds; ch, chloroplasts; fi, fibers; mi, mitochondria; pl, plastoglobuli; sg, starch grains; vc, vacuole; *, subcellular dissolution process. Scale bars: A, E: 50 μm; B-C, F-G: 2 μm; D, H: 1 μm.

Even in the natural environment, different factors, such as water availability during the seasons, can change the anatomy and ultrastructure of *C. cangae* leaves. Visually, in the dry season, the brownish-red leaves exhibit a mesophyll with fewer intercellular spaces, in addition to more cells containing phenolic compounds (Fig 7A). Comparatively, the reddish-green leaves that develop in the rainy season have a mesophyll with conspicuous intercellular spaces, and the number of cells synthesizing phenolic compounds appears reduced (Fig 7E). It is also noted that the fibers of the central vascular bundle assume a darker coloration during the dry season (Fig 7A). Quantitatively, leaves from the dry season show smaller epidermal cells only on the abaxial surface, along with reduced thickness of the lamina, mesophyll, and both palisade and spongy parenchyma (Fig 5).

In the dry season, degradation of chloroplasts occurs, with the disappearance of grana stacking and disruption of the chloroplast envelope; note that the stroma takes on a darker color (Fig 7B-7D). Furthermore, subcellular dissolution is observed in the mesophyll cells in these leaves (Fig 7B-7C – asterisks). On the other hand, leaves that develop during the rainy season have functional cells, observing the integrity of the membranes and organelles (Fig 7F-7H). The chloroplasts are elongated with well-compartmentalized grana stacks (Fig 7F-7G) and stroma with numerous plastoglobuli (Fig 7G-7H). The leaves have cells with a central vacuole and large starch grains (Fig 7B, 7D, 7F–7H-7H) in both seasons (dry and rainy).

## Discussion

The results presented for *C. cangae* indicate the adaptive responses to the xeric conditions of the cangas, as it grows on a rocky substrate with few nutrients, low water retention, high light exposure, daily temperature variations, and winds. Comparative analyses of samples collected under natural and cultivation conditions helped indicate which traits are associated with cangas. Xeromorphic leaf characters are accentuated in the dry season, while the regrowth of new branches and leaves occurs in the rainy season. Thus, developmental aspects and effects of the seasonality are also discussed.

### *Carajasia cangae* possesses a set of adaptive traits to the stressful canga environment

The establishment and development of individuals on rocky outcrops, a water-limited environment, especially during the dry season, support the interpretation that *C. cangae* exhibits xeromorphic features. Here, we highlight that xeromorphic characters and edaphic endemism are associated with water stress and high luminosity/radiation [11]. The results revealed a weakened anatomical organization in etiolated organs grown under cultivation conditions. Specifically, they lacked phenolic pigmentation and showed poorly developed vascular tissues in stems and leaf veins. From an evolutionary point of view, this pattern may be related to the very low genotypic plasticity of the species [20], potentially favoring phenotypic specialization to xeric canga conditions.

Under natural conditions, the rigid stems provide mechanical support on the rocky substrate and winds, in addition to the short stature of the species. Secondary growth contributes to this function, and more significant development of xylem tissues is associated with the efficiency of water transport [34] when water is available. The collapsed arrangement of epidermal and cortex cells, with phenolic compounds, may act to decrease permeability and thus reduce water loss from the internal tissues of the stem. It is worth noting that the synthesis of phenolic compounds is a dynamic process that can be influenced by stressful conditions, including limited water availability, high solar radiation, and the presence of heavy metals in the soil (e.g., [16,35–38]), as well as by the response determined by the species’ genotype [39]. Such stress factors are common in cangas, leading *C. cangae* to produce phenolic compounds, at least in its aboveground vegetative organs. Under water stress in particular, phenolic compounds may act as an antioxidant defense mechanism, reducing cellular damage and protecting the photosynthetic apparatus [36].

The smaller and thicker blades of the individuals under natural conditions are common traits for plants from xeric environments or growing under high radiation conditions [3]. About the cell shape (in frontal view), our results indicate that mesic conditions (under cultivation) contribute to the greater sinuosity of the anticlinal walls, also observed for *Ipomoea* species that grow in cangas after being replanted in a nursery [16]. On the other hand, the leaves of canga plants, under xeromorphic conditions, appear to exhibit reduced sinuosity on the adaxial epidermal surface compared to the abaxial surface (16, our data). Further leaf studies with more canga species could validate this hypothesis.

Considering epidermal papillae, despite being a taxonomic character for Rubiaceae taxa [40–42, our data], different functions are attributed to them. Haberlandt [43] indicates that these papillae function as optical lenses, enhancing light capture and thereby improving photosynthetic performance. The luminosity, which generally has a greater incidence on the adaxial surface of the leaves of *C. cangae*, may explain the greater occurrence of these cells and trichomes [44] on this surface.

Although the leaves of *C. cangae* are amphistomatic, which is common for many Rubiaceae [45], stomata are visually abundant on the abaxial surface. This condition may reduce water loss through transpiration [3], indicating that gas exchange regulation occurs mainly on the abaxial surface of the blade, as in functionally hypostomatic plants [16,46,47].

The mesophyll anatomy of *Carajasia* changes depending on lighting conditions to optimize photosynthesis. A thicker lamina under natural conditions (due to more layers of cells and elongated palisade cells in the mesophyll) promotes more efficient water use and lower transpiration rates under high radiation conditions [3]. In the natural environment, more radiation is involved in developing more mesophyll cells, which are smaller, with those palisades more elongated. This condition facilitates the penetration of light deeper into the mesophyll by the columnar palisade tissue [48]. The lower radiation under cultivation modulates the formation of leaves with fewer cells in the mesophyll, which may represent a lower photosynthetic efficiency per unit area. However, these cells are more voluminous, explaining why the leaves are bigger, and as a mechanism to adjust the light intake. The larger and fewer cells may contribute to increased mesophyll porosity and, consequently, to enhanced CO₂ diffusion within the leaf [49]. Likewise, conspicuous chloroplasts under low radiation respond to adjusting the photosynthetic capacity.

Concerning the vascular system, the fibrous central bundle under natural conditions is remarkable. These fibers promote stability for the tissues and the organ [34]. Vascular bundles are associated with the mucilaginous cells, which delay dehydration due to their ability to retain water [6]. It is worth noting that these cells have a mixed composition, as they also store phenolic compounds. Interestingly, these secretory cells with a mixed composition of mucilaginous and phenolic compounds have already been reported for other canga plants [16].

Our results for plants under natural and cultivation conditions were helpful in elucidating responses to cangas. The results for individuals under natural conditions revealed structural features of vegetative organs that are consistent with traits commonly linked to drought tolerance, in addition to high solar radiation. It is important in a scenario where climate change accelerates and intensifies drought events, adding knowledge on responses by wild plants, considering that these studies have received more attention for crops [48,50].

### Resprouting strategies and developmental issues

Seasonality mediates the life cycle of *C. cangae*, including the loss of aerial mass throughout the dry season. However, the dead leaves remain attached to the stem for a period and resprout in the rainy season. At the stem base, adventitious buds contribute to forming new aerial branches. These branches have reduced lateral branches that develop from axillary buds. All these buds, in the stem base and on the nodes of aerial branches, exhibit regrowth potential [51]. Despite this, belowground bud-bearing organs are indicated as the main strategy for regrowth (e.g., [52–55]), including for Brazilian ecosystems (e.g., Cerrado; [56,57]). In the case of *C. cangae*, which grows on rocky outcrops or in their cracks, the base of the stem appears as a structure that may be partially buried.

The results show that differentiated portions of the main aerial stems and the stem base store carbohydrates as starch grains, in the pith and the vascular parenchyma. Thus, the stem of *C. cangae* appears as a storage organ. Our anatomical data show that the pith of aerial branches is strongly protected by a mechanical barrier (epidermis + cortex) composed of compacted cells with phenolic compounds and by the secondary xylem. Starch storage may be involved in tissue maintenance, regrowth, and development of new branches [4,7,58].

The reduced lateral branches disposed along the aerial main branches are related to flowers and fruit production. Thus, these branches have limited growth, allowing us to characterize them as brachyblasts. Our data showing the opposite arrangement of leaves in significantly reduced internodes of the brachyblasts explains the term “pseudoverticillate leaves” used by Salas et al. [19]. However, these brachyblasts are distinguished by having colleters, which are multicellular structures producing a sticky secretion and associated with the reproductive phase, and/or organs under development (i.e., primordia and young leaves) in angiosperms [3,59]. Immature colleters of *C. cangae* are close to the shoot apical meristem and first leaf primordia, with secretory cells showing a strongly stained cellular content. The mature colleters can be recognized by the presence of phenolic compounds in their parenchymatous cells and the collapsed secretory cells, indicating that they are short-lived during leaf development.

We highlight in the leaf primordia the differentiation of the protodermis/epidermis with papillae, trichomes, and stomata at different developmental stages, and the mesophyll with idioblasts storing phenolic compounds and raphides. The differentiation of numerous stomata is more frequent on the abaxial surface compared to the adaxial surface [60]. During the differentiation process, mechanical coupling between guard cells and surrounding epidermal cells during stomatal movements [61,62] can generate mechanical stresses. Such physical pressures between cells during leaf expansion and epidermal differentiation shape the sinuosity of common epidermal cells [63,64]. This may explain the change from straight-walled or slightly sinuous cells in leaf primordia to highly sinuous-walled cells in fully expanded leaves of *C. cangae*.

Regarding phenolic compounds and raphides, ecological and structural functions are associated with initial stages of leaf development. Both phenolic compounds and raphides may protect the primordia and young leaves against herbivory [65,66]. Furthermore, raphide-containing idioblasts are involved in calcium oxalate storage [17,65,66, our data] and contribute to mass gain in developing tissues, providing support [34,67]. The differentiation of these crystals within spongy parenchyma cells may favor a better distribution of light in deeper layers of the mesophyll [48]. In Rubiaceae, the raphide crystal-type is useful for taxonomy, a synapomorphy for the Spermacoce clade [19]. In the expanded leaves of plants under natural conditions, the synthesis of phenolic compounds is a remarkable characteristic since it also happens later in the epidermis and the vascular sheath cells.

### Seasonally modulated leaf traits explain drought tolerance

Responses of cells and tissues to water loss due to seasonality and cultivation trials are fundamental issues to understanding how *C. cangae* can survive in the cangas. Seasonality changes anatomical aspects of the mesophyll concerning the intercellular spaces, synthesis of phenolic compounds, and resistance of the vascular bundles.

Leaves that expand during the rainy season, with greater water availability in the environment, have visibly increased intercellular spaces in the spongy parenchyma. This condition favors gas exchange, improving photosynthesis [3], which can be even more efficient in thicker leaves during the rainy season, with more developed mesophyll, with both palisade and spongy parenchyma thicker. On the other hand, gas exchange can be detrimental under water stress conditions, such as prolonged drought periods. Thus, leaves that develop a compact mesophyll in the dry season may have better control over water loss during stomatal opening. In these leaves, the synthesis of phenolic compounds intensifies in response to water stress. The synthesis of these compounds explains the red-brownish color of the species during the dry season. Comparably, the vascular bundles become more strongly stained, indicating drought-induced lignin accumulation, as observed for plants subjected to desiccation in controlled experiments [4,6,68].

Leaf ultrastructure also indicates that *C. cangae* is a drought-tolerant species. The results showing the disintegrating cytoplasm is partly derived from the loss of water from the protoplast. It indicates that *C. cangae* tolerates certain levels of dehydration but not desiccation (e.g., resurrection plants) [1], not being a desiccation-tolerant species. Still, regarding the cytoplasm, there is no reorganization of chloroplasts (i.e., dismantled thylakoids) or vacuoles (i.e., a division into smaller or polarization), as usually occurs for many desiccation-tolerant species (e.g., [4,6,69,70]). We observed that in *C. cangae*, a drought-tolerant species, organelles, including the chloroplast and the central vacuole, are disintegrated. These ultrastructural characteristics occurring in the dry season cause irreparable damage with no drought repair mechanisms; such facts do not contemplate survival to desiccation [71]. It should lead the leaf to senescence, which does not occur immediately due to the structure of the central vascular bundle and the greater lignification during the drought period. Before leaf death and late senescence (e.g., marcescent leaves), the synthesis of antioxidant compounds may occur [69], such as the phenolics in *C. cangae*, which are also intensified by high radiation and drought. Collapsed vacuoles, as observed in *C. cangae* leaves in the dry season, release nucleases and proteases into the cytoplasm, causing acidification and contraction of the cytoplasm and the breakdown of DNA, RNA, proteins, and membranes [69], leading to cell death.

The anatomy and ultrastructure of marcescent leaves of a tropical plant were then studied, contributing to the understanding of this leaf condition, whose ecological consequence of maintaining dead biomass aboveground is not well understood [72]. Functionally, marcescent leaves originate from leaves with prolonged photosynthetic activity that suddenly die at the very end of the season [73], as seen with *C. cangae* at the end of the dry season. In this context, these dead leaves attached to the stem protect the axillary buds, which will resprout in the rainy season.

The rainy season should be in dialogue with the plants cultivated. However, light conditions impose differences on specific traits. In the rainy season, the leaf mesophyll presents cells with a large central vacuole, intact organelles, including chloroplasts with well-compartmentalized grana stacks facing the inner surface of the cell walls; this arrangement is related to better luminosity perception (see 48) in the functional leaves of *C. cangae*. Chloroplasts still have many plastoglobuli, which can prevent photooxidative damage [6,69] in photosynthetically active leaves.

All the results obtained – morphology, anatomy, and ultrastructure – indicate that *C. cangae* leaves have a certain plasticity under natural conditions of the cangas, but not under the cultivation conditions tested.

## Conclusions

*Carajasia cangae* proved to be an interesting model to indicate structural responses to the xeric conditions of the cangas. The larger individuals are drought-tolerant and capable of resprouting during the rainy season. The persistence of *C. cangae* throughout the seasons occurs due to a set of xeromorphic structural characters, which were identified by comparison between plants under natural and cultivated conditions. Under natural conditions, the stem is rigid, with compacted epidermis and cortex, and differentiated secondary vascular tissues. The leaves stand out for their thicker mesophyll, with more elongated palisade cells, and for the fibrous central vascular bundle. Cultivated plants, on the other hand, exhibit drastic changes, indicating that the species is highly dependent on the ferruginous substrate and high radiation inherent to its natural habitat. Seasonality modulates leaf morphostructural adjustments and ultrastructure, with no repair desiccation mechanisms, with the intensification of the dry period. All of the responses help to explain how adult individuals persist on rocky and ferruginous outcrops.

## Supporting information

S1 File Carajasia cangae Measurements for statistics. (XLSX)
